# Obesity and Occupational Injury: A Prospective Cohort Study of 69,515 Public Sector Employees

**DOI:** 10.1371/journal.pone.0077178

**Published:** 2013-10-16

**Authors:** Anne Kouvonen, Mika Kivimäki, Tuula Oksanen, Jaana Pentti, Roberto De Vogli, Marianna Virtanen, Jussi Vahtera

**Affiliations:** 1 School of Sociology, Social Policy and Social Work, Queen’s University Belfast, Belfast, United Kingdom; 2 UKCRC Centre of Excellence for Public Health (Northern Ireland), Queen’s University Belfast, Belfast, United Kingdom; 3 Finnish Institute of Occupational Health, Turku and Helsinki, Finland; 4 Department of Epidemiology and Public Health, University College London, London, United Kingdom; 5 Institute of Behavioral Sciences, University of Helsinki, Helsinki, Finland; 6 Department of Public Health Sciences, School of Medicine, University of California, Davis, United States of America; 7 Department of Public Health, University of Turku, and Turku University Hospital, Turku, Finland; Old Dominion University, United States of America

## Abstract

**Background:**

Obesity and overweight are suggested to increase the risk of occupational injury but longitudinal evidence to confirm this is rare. We sought to evaluate obesity and overweight as risk factors for occupational injuries.

**Methodology/Principal Findings:**

A total of 69,515 public sector employees (80% women) responded to a survey in 2000–2002, 2004 or 2008. Body mass index (kg/m^2^) was derived from self-reported height and weight and was linked to records of subsequent occupational injuries obtained from national registers. Different injury types, locations and events or exposures (the manner in which the injury was produced or inflicted) were analyzed by body mass index category adjusting for baseline socio-demographic characteristics, work characteristics, health-risk behaviors, physical and mental health, insomnia symptoms, and sleep duration. During the mean follow-up of 7.8 years (SD = 3.2), 18% of the employees (*N* = 12,204) recorded at least one occupational injury. Obesity was associated with a higher overall risk of occupational injury; multivariable adjusted hazard ratio (HR) 1.21 (95% CI 1.14–1.27). A relationship was observed for bone fractures (HR = 1.37; 95% CI: 1.10–1.70), dislocations, sprains and strains (HR = 1.36; 95% CI: 1.25–1.49), concussions and internal injuries (HR = 1.26; 95% CI: 1.11–1.44), injuries to lower extremities (HR = 1.62; 95%: 1.46–1.79) and injuries to whole body or multiple sites (HR = 1.37; 95%: 1.10–1.70). Furthermore, obesity was associated with a higher risk of injuries caused by slipping, tripping, stumbling and falling (HR = 1.55; 95% CI: 1.40–1.73), sudden body movement with or without physical stress (HR = 1.24; 95% CI: 1.10–1.41) and shock, fright, violence, aggression, threat or unexpected presence (HR = 1.33; 95% CI: 1.03–1.72). The magnitude of the associations between overweight and injuries was smaller, but the associations were generally in the same direction as those of obesity.

**Conclusions/Significance:**

Obese employees record more occupational injuries than those with recommended healthy weight.

## Introduction

Increased prevalence of overweight and obesity is a major public health concern. In over half of the Organisation for Economic Co-operation and Development (OECD) countries at least 50% of adults are now overweight or obese. [1] In Finland 20% of adults are obese; this is higher than the OECD average (17%). [1] Obesity is a known risk factor for a number of diseases, including diabetes, coronary heart disease, stroke, cancer, and osteoarthritis. [2] Although obesity is common in working-age populations, its effect on occupational injuries remain unclear.

A systematic review of 12 studies published between 1980 and 2005 concluded that overall the risk of injury was slightly increased for obese employees; however, there were some inconsistencies between the reviewed studies. [3] In recent cross-sectional studies of representative samples of Canadian [4] and US [5] working populations, obese workers were more likely to report occupational injuries, but no increased risk was observed for overweight workers. Obesity was associated with occupational injury, particularly with knee and leg injuries, in a large prospective study of manufacturing employees in the US. [6] However, most of the previous studies have been limited in terms of their cross-sectional design, small sample sizes, and/or failure to control for potentially important confounders and examine effect modification. Large-scale prospective studies are rare. In addition, it has remained unclear whether the risks differ depending on the type, location, and mechanism of occupational injury. [4], [6], [7].

In this prospective study of nearly 70,000 male and female employees, we sought to examine obesity and overweight as predictors of recorded occupational injuries controlling for a range of potential confounding factors. The large sample size enabled us to examine effect modification in relation to employee sex, age and socioeconomic status (SES), which has not previously been feasible. Furthermore, we investigated whether the associations are different in relation to types, anatomical sites, and the manners in which occupational injuries were produced or inflicted; all of which there is very little previous literature**.** To our knowledge, this is the largest prospective observational cohort study on obesity and occupational injuries to date.

## Materials and Methods

The study population comprised the participants of the Finnish Public Sector Study, which is an ongoing prospective epidemiological cohort study of employees working in 10 towns and 21 hospitals in six hospital districts. [8] We included all participants who responded to questionnaire surveys either in 2000–2002 (*N* = 48,598, response rate 68%), 2004 (*N* = 48,076, response rate 66%) or 2008 (*N* = 52,891, response rate 71%). In case of repeat responses, we used the earliest response in the analyses (79% of the 99,699 eligible employees responded at least once). We excluded 8,749 participants with missing data on height, weight, or covariates and 52 survey respondents who either died or retired before the follow-up began, that is, immediately after the survey response. The final analytic sample comprised 69,515 participants with complete baseline information. The final sample did not substantially differ from the eligible population in terms of mean age (43.5 years in the sample, 42.8 years in the eligible population) or the proportion of women (80% vs. 77%). The sex and age distribution of the participants is representative of Finnish public sector employees.

In Finland, occupational injuries are compensated through a statutory insurance system. Using personal identification numbers (unique number assigned to all Finnish residents), the respondents were linked to comprehensive national occupational injury registers maintained by the Federation of Accident Insurance Institutions. Deterministic approach was used to link the data. The record linkage was successful for all participants.

### Ethics Statement

The study was approved by the Ethics Committee of Hospital District of Helsinki and Uusimaa. The use of a questionnaire acts as a form of written informed consent. All data were analyzed anonymously.

### Occupational Injury

In Finland, the employer is obliged to purchase a statutory policy for the employees to cover occupational injuries. Moreover, compensation for occupational injuries takes priority over other forms of statutory compensation and pensions, and, for example, medical treatment expenses are fully covered. The study outcomes were the occurrence of any recorded occupational injury between the date of the survey response and December 31, 2011 (mean follow-up time 7.8, SD 3.2 years). By definition, occupational injury is an injury to the employee caused by an accident due to an unexpected, sudden external event.

Information on the type of the occupational injury, the primary body part injured (anatomical site), and the manner in which the injury was produced or inflicted was collected using the Federation of Accident Insurance Institutions classifications and combining the categories in which numbers were small. The occupational injury types were the following: 1) wounds and superficial injuries; 2) bone fractures; 3) dislocations, sprains and strains; 4) concussions and internal injuries; 5) burns, scalds and frostbites; 6) poisonings and infections, drowning and asphyxiation; 7) other, multiple injuries. In the analysis we combined the last three categories, because the numbers of cases in these categories were small.

The categories of anatomical sites of injuries were as follows: 1) head; neck, including spine and vertebra in the neck, torso; 2) back, including spine and vertebra in the back; 3) upper extremities; 4) lower extremities; 5) other parts, whole body and multiple sites.

According to the Occupational Injury and Illness Classification System (OIICS), “the event or exposure describes the manner in which the injury or illness was produced or inflicted by the source of injury or illness”. [9] The event/exposure had the following categories: 1) Breakage, fall, collapse of material agent; 2) Loss of control of machine, means of transport, handling equipment, handheld tool, animal; 3) Slipping, tripping, stumbling and falling; 4) Contact with sharp material agent; 5) Sudden body movement with or without physical stress; 6) Shock, fright, violence, aggression, threat, unexpected presence; 7) Other.

### Overweight and Obesity

Body mass index (BMI) [(weight (kilograms) divided by height (meters) squared)], which is the most commonly used indicator of overweight and obesity in adults, was calculated based on self-reported weight and height. BMI was categorized for each employee according to standard thresholds:<18.5 kg/m^2^ underweight; 18.5–24.9 kg/m^2^ recommended healthy weight; 25.0–29.9 kg/m^2^ overweight; ≥30 kg/m^2^ obesity. [10].

### Baseline Covariates

A number of covariates were included in the analyses as potential confounders. All covariates were measured at baseline. Socio-demographic and work-related data from the employers’ registers included information of the participants’ age, sex, educational attainment (less than higher education vs. higher education), occupational status, and type of job contract (fixed term vs. permanent). Occupational status was used as an indicator of socioeconomic status (SES) and was split into three categories: manual (e.g., cleaners, maintenance workers), lower-grade non-manual (e.g., registered nurses, technicians) and higher-grade non-manual (e.g., teachers, physicians); according to the Classification of Occupations by Statistics Finland, [11] as in previous studies. [12] Marital status (married or co-habiting vs. not) and work schedule (night/shift work vs. day work) were obtained from the surveys.

Information on health-risk behaviors was derived from survey responses. Participants reported their average weekly consumption of beer, wine, and spirits. The reported amounts were converted into grams of pure alcohol and >210 g of pure alcohol per week was used as a cut-off for heavy drinking (no/yes). [13] Participants assessed the quantity of their physical activity equivalent to walking, brisk walking, jogging, or running. Low physical activity was defined as ≤2 Metabolic Equivalent Task hours per day (no/yes). [14].

Self-rated health status was classified as sub-optimal (average or worse) or optimal (good or very good health). [15] Psychological distress (no/yes) was evaluated by the 12-item version of the General Health Questionnaire (GHQ-12). [16] Cut-off was a report of psychological distress in at least four items.

The 4-item Jenkins Sleep Problem Scale was used to measure self-reported insomnia symptoms. [17] The scale includes four dimensions corresponding to the diagnostic criteria of insomnia (DSM-IV): difficulties initiating sleep, waking up several times per night, too early morning awakenings, and non-refreshing sleep. Participants rated on a scale from 1 = “never” to 6 = “nearly every night” to what extent they had experienced the symptoms during the past four weeks. If the participant reported more than one symptom, their most frequent symptom was used to assess the degree of their symptoms. Insomnia symptoms were dichotomised as follows: no or moderate insomnia symptoms (up to 4 nights/week) vs. severe insomnia symptoms (5 to 7 nights/week). In addition, the usual sleep duration (per 24 hours) was measured by self-reports and split into three categories: 6.5 hours or less; 7 to 9.5 hours; 9 hours or more.

### Statistical Analysis

The associations between baseline covariates and BMI categories were analyzed using the Chi-square test. Cox proportional hazard models were used to examine the association between baseline BMI categories and injuries during the follow-up. We calculated hazard ratios (HRs) and their 95% confidence intervals (CI) for occupational injuries (overall, specific types, specific locations, and the event or exposure preceding the injury). Follow-up began from the date of the survey response and ended at the first occurrence of the outcome measure, retirement, death, or on 31 December, 2011, whichever came first.

Adjustments were made for potential confounders: age, sex, education, SES, marital status, type of job contract, night/shift work, health-risk behaviors, self-rated health, psychological distress, self-reported insomnia symptoms, and sleep duration. Sex and SES differences were examined by including the interaction terms “age × BMI categories“ (in models including the main effects and adjusted for sex), “sex × BMI categories” (in models including the main effects and adjusted for age), and “SES × BMI categories” (in models including the main effects and adjusted for age and sex).

The SAS statistical software, version 9.3, was used to conduct the analysis (SAS Institute, Inc., Cary, North Carolina).

## Results

Over half (55%) of the employees in this sample had BMI in the recommended healthy range. About a third (32%) of the participants were overweight and another 12% were categorized as obese. Only 1.2% of the participants were underweight. The mean BMI was 25.1 (SD = 4.2, range 15.0–49.9). Older employees were more often overweight or obese than those younger than 40 years of age. In addition, male participants, the less educated, manual workers, those who were married or cohabiting, employees working nights or shifts and employees with permanent job contract were more often above healthy weight, as were heavy drinkers, current smokers, those with low physical activity, those reporting psychological distress, suboptimal self-rated health, severe insomnia symptoms, and those who slept on average only 6.5 hours or less per night (all *p* values<0.001) (Table 1).

**Table 1:** Characteristics of the study participants at baseline (*N* = 69,515).

		*Body Mass Index Status*			
*Characteristic*	*N* (%)	Underweight*N* (%)	Healthy weight*N* (%)	Overweight*N* (%)	Obese*N* (%)
Sex					
Women	55,240 (80)	825 (2)	32,262 (58)	15,654 (28)	6499 (12)
Men	14,275 (20)	32 (0)	5718 (40)	6589 (46)	1936 (14)
Age (years)					
17 to 39	24,348 (35)	500 (2)	15,419 (63)	6221 (26)	2208 (9)
40 to 49	23,386 (34)	236 (1)	12,765 (55)	7568 (32)	2817 (12)
50 to 67	21,781 (31)	121 (1)	9796 (45)	8454 (39)	3410 (16)
Education					
Less than higher education	29,988 (43)	332 (1)	14,199 (47)	10,757 (36)	4700 (16)
Higher education	39,527 (57)	525 (1)	23,781 (60)	11,486 (29)	3735 (10)
Socio-economic status (SES)					
High	21,259 (31)	288 (1)	12,794 (60)	6330 (30)	1847 (9)
Intermediate	36,119 (52)	457 (1)	20,191 (56)	11,026 (31)	4445 (12)
Manual	12,137 (17)	112 (1)	4995 (41)	4887 (40)	2143 (18)
Married or cohabiting					
Yes	52,644 (76)	567 (1)	28,497 (54)	17,257 (33)	6323 (12)
No	16,871 (24)	290 (2)	9483 (56)	4986 (30)	2112 (13)
Type of job contract					
Permanent	56,063 (81)	579 (1)	29,699 (53)	18,698 (33)	7087 (13)
Fixed term	13,452 (19)	278 (2)	8281 (62)	3545 (26)	1348 (10)
Night/shift work					
No	45,891 (66)	561 (1)	25,435 (55)	14,441 (31)	5454 (12)
Yes	23,624 (34)	296 (1)	12,545 (53)	7802 (33)	2981 (13)
Current smoking					
No	56,900 (82)	658 (1)	31,414 (55)	18,043 (32)	6785 (12)
Yes	12,615 (18)	199 (2)	6566 (52)	4200 (33)	1650 (13)
Heavy drinking					
No	63,784 (92)	815 (1)	35,477 (56)	19,926 (31)	7566 (12)
Yes	5731 (8)	42 (1)	2503 (44)	2317 (40)	869 (15)
Insufficient physical activity					
No	52,434 (75)	677 (1)	30,609 (58)	16,112 (31)	5036 (10)
Yes	17,081 (25)	180 (1)	7371 (43)	6131 (36)	3399 (20)
Psychological distress					
No	52,216 (75)	644 (1)	28,728 (55)	16,745 (32)	6089 (12)
Yes	17,299 (25)	213 (1)	9242 (53)	5498 (32)	2346 (14)
Suboptimal self-rated health					
No	52,747 (76)	713 (1)	31,357 (60)	15,785 (30)	4892 (9)
Yes	16,768 (24)	144 (1)	6623 (40)	6458 (39)	3543 (21)
Insomnia symptoms					
No or moderate	54,027 (78)	682 (1)	30,164 (56)	17,099 (32)	6082 (11)
Severe	15,488 (22)	175 (1)	7816 (51)	5144 (33)	2353 (15)
Sleep duration					
6.5 hours/less	15,388 (22)	174 (1)	7558 (49)	5359 (35)	2297 (15)
7 to 8.5 hours	51,727 (74)	645 (1)	29,088 (56)	16,165 (31)	5829 (11)
9 hours or more	2400 (4)	38 (2)	1334 (56)	719 (30)	309 (13)

*P*<0.0001 in all cases.

A total of 12,204 employees (18%) experienced at least one occupational injury during the mean follow-up of 7.8 years. Only 4% of employees sustained more than one injury. Dislocations, sprains and strains (41% of all injuries) were the most frequent type of injury, and upper extremities (36%) were the most common injury location, and slipping, tripping, stumbling and falling (25%) were the most common events or exposure categories.

Sixteen percent of those with recommended healthy weight, 17% of those who were underweight, 19% of those who were overweight and 21% of those who were obese had sustained an occupational injury during the follow-up. A higher proportion of occupational injuries was observed for manual workers (33%) than for lower (17%) or higher-grade (11%) non-manual employees. Men (25%) were more prone to injuries than women (16%). Employees aged 17 to 39 years of age (18%) and those aged 40 to 49 years (19%) had sustained an occupational injury more often than employees aged over 50 (15%).

**Figure 1:**
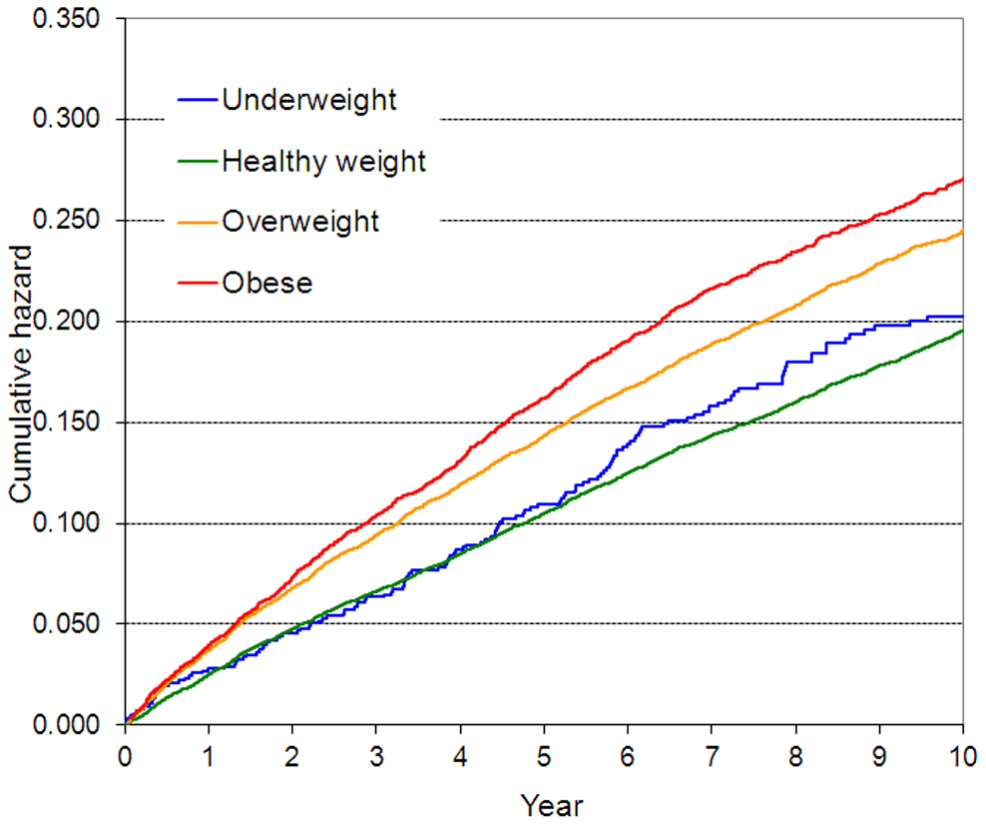
Cumulative hazard for occupational injury by BMI categories.

Table 2 shows that being obese or overweight was associated with an increased risk of occupational injury. Unadjusted HRs were 1.51 (95% CI: 1.43–1.59) for obese participants and 1.33 (95% CI: 1.28–1.38) for those categorized as overweight, in comparison to those with recommended healthy weight. These associations attenuated but remained significant after adjustment for potential confounders: age, sex, marital status, education, SES, type of job contract, night/shift work, health-risk behaviors, psychological distress, self-rated health, insomnia symptoms, and sleep duration (HR = 1.21; 95% CI: 1.14–1.27 for obese employees and HR = 1.13; 95% CI: 1.08–1.18 for overweight employees). Figure 1 shows the cumulative hazard curves for occupational injury in different BMI categories.

**Table 2:** Association between BMI status and occupational injury: the Finnish Public Sector Study (*N* = 69,515).

BMI status	*N*/cases	Model 1HR (95% CI)	Model 2HR (95% CI)	Model 3HR (95% CI)
Underweight	857/143	1.03 (0.88–1.22)	1.05 (0.89–1.24)	1.04 (0.88–1.23)
Healthyweight	37,980/6045	1.00	1.00	1.00
Overweight	22,243/4285	1.33 (1.28–1.38)	1.14 (1.09–1.18)	1.13 (1.08–1.18)
Obese	8435/1731	1.51 (1.43–1.59)	1.23 (1.17–1.30)	1.21 (1.14–1.27)
*P* for linear trend		<0.0001	<0.0001	<0.0001

BMI, body mass index; HR, hazard ratio; CI, confidence interval. Model 1 unadjusted. Model 2 adjusted for age, sex, marital status, education, socio-economic status, type of job contract, and night/shift work. Model 3 as Model 2, additionally adjusted for health-risk behaviors (smoking, heavy drinking, insufficient physical activity), psychological distress, self-rated health, insomnia symptoms, and sleep duration.

The interactions age × BMI categories (*p*<0.0001) and SES × BMI categories (*p* = 0.016) were significant at *p*<0.05 level, whereas the interaction between sex × BMI categories was not (*p* = 0.275). Consequently, we conducted age- and SES-stratified analyses. Age-stratified models showed that obesity was slightly more strongly associated with an injury risk in employees aged 17 to 39 (HR = 1.32; 95% CI: 1.20–1.46) than in the second oldest age group (40 to 49 years) (HR = 1.20; 95% CI: 1.10–1.31). No association between obesity and occupational injury was observed in the oldest age group (50 to 67 years) (HR = 1.08; 95% CI: 0.98–1.20) (Table 3).

**Table 3:** Association between BMI status and occupational injury by age group: the Finnish Public Sector Study (*N* = 69,515).

BMI status	17 to 39 years(*N* = 24,348)	40 to 49 years(*N* = 23,386)	50 to 67 years(*N* = 21,781)
	HR (95% CI)	HR (95% CI)	HR (95% CI)
Underweight	1.09 (0.88–1.36)	0.94 (0.68–1.30)	1.23 (0.80–1.90)
Healthy weight	1.00	1.00	1.00
Overweight	1.20 (1.12–1.29)	1.12 (1.04–1.19)	1.05 (0.97–1.13)
Obese	1.32 (1.20–1.46)	1.20 (1.10–1.31)	1.08 (0.98–1.20)
*P* for linear trend	<0.0001	<0.0001	0.134

BMI, body mass index; HR, hazard ratio; CI, confidence interval. Adjusted for sex, marital status, education, socio-economic status, type of job contract, and night/shift work, smoking, heavy drinking, insufficient physical activity, psychological distress, self-rated health, insomnia symptoms, and sleep duration.

SES-stratified models are presented in Table 4. Obesity was more strongly associated with occupational injury risk in lower-grade (HR = 1.26; 95% CI: 1.17–1.37) and higher-grade non-manual (HR = 1.26; 95% CI: 1.09–1.47) employees than in manual workers (HR = 1.11; 95% CI: 1.02–1.22).

**Table 4:** Association between BMI status and occupational injury by socio-economic group: the Finnish Public Sector Study (*N* = 69,515).

BMI status	Manual workers (*N* = 12,137)	Lower-grade non-manualemployees (*N* = 36,119)	Higher-grade non-manual employees (*N* = 21,259)
	HR (95% CI)	HR (95% CI)	HR (95% CI)
Underweight	0.74 (0.50–1.10)	1.13 (0.90–1.40)	1.14 (0.82–1.59)
Healthy weight	1.00	1.00	1.00
Overweight	1.10 (1.03–1.18)	1.13 (1.06–1.20)	1.19 (1.08–1.31)
Obese	1.11 (1.02–1.22)	1.26 (1.17–1.37)	1.26 (1.09–1.47)
*P* for linear trend	0.0024	<0.0001	0.0002

BMI, body mass index; HR, hazard ratio; CI, confidence interval. Adjusted for age, sex, marital status, education, type of job contract, and night/shift work, smoking, heavy drinking, insufficient physical activity, psychological distress, self-rated health, insomnia symptoms, and sleep duration.

In the total analytic sample, higher than recommended BMI increased the risk of only some types of occupational injuries (Table 5). In fully adjusted models, obesity was associated with an increased risk of bone fractures (HR = 1.37; 95% CI: 1.11–1.70), dislocations, sprains and strains (HR = 1.36; 95% CI: 1.25–1.49), and concussions and internal injuries (HR = 1.26; 95% CI: 1.10–1.44). Compared to employees with BMI in the recommended healthy range, overweight employees also had a higher risk of dislocations, sprains and strains (HR = 1.23; 95% CI: 1.15–1.31) and concussions and internal injuries (HR = 1.19; 95% CI: 1.08–1.31). In terms of the anatomical sites, obesity was associated with an increased risk of injuries to upper extremities (HR = 1.11; 95% CI: 1.01–1.22), lower extremities (HR = 1.62; 95% CI: 1.46–1.79), and injuries to the whole body or multiple locations (HR = 1.37; 95% CI: 1.10–1.70). Being overweight increased the risk of injuries to back, lower extremities, and whole body/multiple locations; in the fully adjusted models the HRs varied between 1.14 (95% CI: 1.02–1.27) and 1.27 (95% CI: 1.17–1.37).

**Table 5:** Associations between BMI status and different types and anatomical sites of occupational injury: the Finnish Public Sector Study (*N* = 69,515).

Occupational injury	*N*	Underweight	Healthy weight	Overweight	Obese
*Type of injury*		HR (95% CI)		HR (95% CI)	HR (95% CI)
Wounds and superficial injuries	3351	1.04 (0.76–1.41)	1.00	1.00 (0.92–1.08)	0.98 (0.88–1.09)
Bone fractures	750	1.45 (0.80–2.65)	1.00	1.05 (0.89–1.24)	1.37 (1.11–1.70)
Dislocations, sprains and strains	5022	1.11 (0.86–1.44)	1.00	1.23 (1.15–1.31)	1.36 (1.25–1.49)
Concussions and internal injuries	2098	0.65 (0.39–1.08)	1.00	1.19 (1.08–1.31)	1.26 (1.10–1.44)
Other or multiple injuries	733	0.98 (0.50–1.90)	1.00	1.04 (0.89–1.23)	0.97 (0.77–1.23)
*Anatomical site of injury*					
Head	1398	1.22 (0.78–1.93)	1.00	1.07 (0.95–1.21)	0.85 (0.71–1.02)
Neck, including spine and vertebrain the neck, Torso	528	1.83 (0.97–3.45)	1.00	0.99 (0.82–1.21)	1.12 (0.86–1.46)
Back, including spine and vertebrain the back	1667	1.27 (0.86–1.89)	1.00	1.14 (1.02–1.27)	0.99 (0.84–1.16)
Upper Extremities	4344	0.89 (0.67–1.19)	1.00	1.05 (0.98–1.13)	1.11 (1.01–1.22)
Lower Extremities	3366	0.78 (0.53–1.14)	1.00	1.27 (1.17–1.37)	1.62 (1.46–1.79)
Other parts, Whole body andmultiple sites	752	1.32 (0.70–2.48)	1.00	1.24 (1.05–1.46)	1.37 (1.10–1.70)
*Event or exposure*					
Breakage, fall, collapse of material agent	880	1.17 (0.66–2.07)	1.00	1.01 (0.86–1.17)	0.94 (0.76–1.17)
Loss of control of machine, meansof transport, handling equipment,handheld tool, animal	716	0.96 (0.48–1.94)	1.00	1.14 (0.96–1.34)	1.19 (0.94–1.50)
Slipping, tripping, stumbling and falling	2986	1.00 (0.70–1.44)	1.00	1.27 (1.17–1.37)	1.55 (1.40–1.73)
Contact with sharp material agent	1634	0.68 (0.40–1.15)	1.00	0.99 (0.88–1.11)	1.02 (0.87–1.19)
Sudden body movementwith or without physical stress	2491	1.08 (0.75–1.54)	1.00	1.19 (1.08–1.30)	1.24 (1.10–1.41)
Shock, fright, violence,aggression, threat,unexpected presence	616	1.37 (0.75–2.50)	1.00	1.30 (1.09–1.56)	1.33 (1.03–1.72)
Other	974	1.08 (0.62–1.88)	1.00	0.99 (0.86–1.14)	1.08 (0.89–1.32)

BMI, body mass index; HR, hazard ratio; CI, confidence interval. Adjusted for age, sex, marital status, education, socio-economic status, type of job contract, and night/shift work, smoking, heavy drinking, insufficient physical activity, psychological distress, self-rated health, insomnia symptoms, and sleep duration.

In terms of the manner in which the injury was produced or inflicted, obesity was associated with an increased risk of injuries caused by slipping, tripping, stumbling and falling (HR = 1.55; 95% CI: 1.40–1.73), sudden body movement with or without physical stress (HR = 1.24; 95% CI: 1.10–1.41) and shock, fright, violence, aggression, threat or unexpected presence (HR = 1.33; 95% CI: 1.03–1.72).

## Discussion

In the present prospective cohort study of nearly 70,000 public sector employees, higher than recommended BMI predicted occupational injuries in a dose–response manner. Compared to employees whose BMI was in the recommended healthy range, overweight employees had a 14% to 30% excess risk and obese employees had a 11% to 62% excess risk of occupational injury. This association was not attributable to potential confounders, such as socio-demographic and work-related characteristics, health-risk behaviors, physical or mental health, insomnia symptoms, or usual sleep duration. Our results are in line with previous studies that have suggested a link between obesity and occupational injury.[4]–[6], [18], [19].

Several possible pathways by which obesity could increase the occupational injury risk have been discussed in the literature. [3], [4] First, daytime consequences of sleep apnea and sleepiness, as well as overall fatigue, which are common in obese people, may contribute to the risk of occupational injury. [20] Second, obese employees have generally poorer health than people with recommended healthy weight and consequently, more often use prescription drugs, which in turn may increase the injury risk. However, in the current study, the adjustment for self-reported overall and mental health, insomnia symptoms and sleep duration affected the results very little.

Third, excess weight can hinder gait and physical functioning and thereby increase the risk of occupational injury. Supporting this notion, the most common injuries related to obesity were dislocations, sprains and strains, bone fractures, and injuries to lower extremities; and in terms of the event or exposure, obesity was associated with an increased risk of slipping, tripping, stumbling and falling and sudden body movement with or without physical stress. Finally, excess body fat could affect the ability of the body to tolerate hazardous mechanical energy exposure.

Only few previous studies have measured different types, locations and mechanisms of occupational injuries and the categorization has not been uniform. However, in line with previous studies, we found that obesity was associated with a higher risk of dislocations, sprains and strains [4], [7], injuries to lower extremities, [4], [6], [7] and upper extremities. [7] Unlike an earlier study [4] we found that obesity was additionally associated with bone fractures. Indeed, accumulating data suggest that obesity is detrimental to bone health, [21] and obesity has been linked to inferior bone quality and markedly lower bone formation. [22] Some evidence suggests that in adults the effects of fat mass on bone and fracture risk may vary by skeletal site: obesity appears to protect against hip and vertebral fractures whilst it is a risk factor for fractures of the humerus and ankle. [23] In line with this, our results showed an increased risk of injuries to lower and upper extremities. Moreover, in our study, overweight and obese employees had a higher risk of concussions and internal injuries, and injuries to the whole body or multiple sites; as far as we are aware, these associations have not been examined in previous studies.

Being overweight was weakly associated with a risk of back injuries. In a systematic review which included five studies that investigated the relationship between obesity and back injury, [3] only one small study reported a significant association with BMI. [24] In a study on obesity and workers’ compensation, claims relating to back were significantly associated with BMI category; with employees with 25<BMI<35 or ≥40 having a higher risk. [7].

In earlier cross-sectional studies the association between of obesity and injury risk has been strongest in older workers. [4], [25] In contrast, in the present study obesity was more strongly associated with injury risk in employees younger than 40 years of age, however, the confidence intervals were overlapping.

In the present study, the relationship between obesity and injury risk was slightly stronger in non-manual than in manual employees. This is in line with an earlier study in a representative sample of Canadian employees which showed that obese employees employed in sedentary occupations were particularly vulnerable to occupational injuries. [4] It has been argued that modern office workers who do not take any exercise during their leisure time have become physically so inactive that they are putting their health at risk. [26] Insufficient physical activity in office work can lead to loss of physical capacity and muscle strength and disturbances in physiological adaptability; [26] these in turn may increase occupational injury risk. Modern office work is usually below the required physical stress thresholds to prevent loss of capacity of in terms of maintenance of muscle strength and enhancing bone mineral density. [26] These problems may occur particularly in sedentary overweight and obese employees who also often have very low physical activity levels in their leisure time.

### Strengths and Limitations

The key strengths of this study are its prospective design with a mean follow-up of nearly eight years and the data derived from a large occupational cohort, which were successfully linked to comprehensive national injury registers with no loss to follow-up. In Finland, the employer is obliged to purchase a statutory insurance policy for the employees to cover occupational injuries. By using the Federation of Accident Insurance Institutions’ national database we were able to determine injury cases based on medical evidence, to detect the exact timing of the injury, and thereby to take temporal issues into account in the analysis. Because of the record-linkage, there was no loss to follow-up.

Our sample was diverse in terms of sex, age, educational levels, and occupational groups; ranging from lower-risk office jobs to more risk-prone manual work. A further strength is that we assessed specific types and locations of injury, examined the relationship between obesity and injury by age and SES, and simultaneously included a number of covariates.

However, several limitations need to be taken into account when interpreting the findings.

First, we used self-reported BMI; earlier research indicates that self-reported weight can be underestimated. [27] However, although particularly overweight and obese people tend to underestimate their weight, a strong agreement exists between self-reported and measured BMI, [28] and the measurement of obesity based on self-reported weight and height is considered to be reasonably accurate. [29].

Second, women were over-represented in the current sample; however the sex distribution was in line with that of the public sector personnel population in Finland and the number of men in our sample was more than 14,000. More diverse samples representing also the private sector are needed to further confirm our findings.

Third, although adjusted for a large number of covariates, it is still possible that confounding due to unobserved variables, such as safety training or risk-taking, remains. [30], [31] However, we adjusted for many of the potential determinants of occupational injuries, including alcohol consumption, physical activity, insomnia, sleep duration, which can be considered not only confounders but also plausible mediators of the association of interest. In the latter case, it can be argued that we have over-adjusted our models.

Finally, the underlying cause of injury is likely to be multifactorial: the environment outside of work, the worker’s mental and physical health, as well as the job and work environment may all play a part. [7] This is an inherent problem in any study looking at occupational injury. [7].

## Conclusions

This prospective study suggests that obesity and overweight increase the risk of occupational injuries. Obese employees seem to be particularly vulnerable to bone fractures, dislocations, sprains and strains, concussions and internal injuries; as well as injuries to upper and lower extremities, and injuries to the whole body and multiple locations. In addition, overweight employees had an increased risk for dislocations, sprains and strains, concussions and internal injuries; as well as injuries to lower extremities, and injuries to back, the whole body and multiple locations. Many of these injury types are often associated with locomotion, for example, lifting or falling. Indeed, in terms of the event or exposure, obesity was shown to be associated with an increased risk of slipping, tripping, stumbling and falling and sudden body movement with or without physical stress. The association between obesity and injury was stronger in employees younger than 40 years of age and in those with non-manual occupations. Weight management and prevention of obesity at the workplace may provide an additional benefit of improving occupational injury rates. There is some evidence of the positive effect of workplace-based weight loss programs on workplace injuries in male shift workers. [32] Future studies are needed to confirm whether weight management interventions at the workplace will have a potential to improve occupational injury rates also in other employee populations. However, obesity alone is unlikely to be a necessary or a sufficient risk factor for occupational injury; therefore reducing weight in obese employees would not eliminate occupational injuries.
